# Unravelling the microbiome of wild flowering plants: a comparative study of leaves and flowers in alpine ecosystems

**DOI:** 10.1186/s12866-024-03574-0

**Published:** 2024-10-19

**Authors:** Dinesh Kumar Ramakrishnan, Franziska Jauernegger, Daniel Hoefle, Christian Berg, Gabriele Berg, Ahmed Abdelfattah

**Affiliations:** 1 Leibniz Institute for Agricultural Engineering and Bioeconomy (ATB), Max-Eyth Allee 100, 14469, Potsdam, Germany; 2 Institute of Environmental Biotechnology, Graz University of Technology, Petersgasse 12, 8010 Graz, Austria; 3 Institute of Biology, Department of Plant Sciences, NAWI Graz, University of Graz, 8010 Graz, Austria; 4 Institute for Biochemistry and Biology, University of Potsdam, 14476 Potsdam, Germany

**Keywords:** Core microbiome, Co-evolution, Phyllosphere, Altitude microbiome, Phylosymbiosis, Pristine microbiome

## Abstract

**Background:**

While substantial research has explored rhizosphere and phyllosphere microbiomes, knowledge on flower microbiome, particularly in wild plants remains limited. This study explores into the diversity, abundance, and composition of bacterial and fungal communities on leaves and flowers of wild flowering plants in their natural alpine habitat, considering the influence of environmental factors.

**Methods:**

We investigated 50 wild flowering plants representing 22 families across seven locations in Austria. Sampling sites encompassed varied soil types (carbonate/silicate) and altitudes (450–2760 m). Amplicon sequencing to characterize bacterial and fungal communities and quantitative PCR to assess microbial abundance was applied, and the influence of biotic and abiotic factors assessed.

**Results:**

Our study revealed distinct bacterial and fungal communities on leaves and flowers, with higher diversity and richness on leaves (228 fungal and 91 bacterial ASVs) than on flowers (163 fungal and 55 bacterial ASVs). In addition, *Gammaproteobacteria* on flowers and *Alphaproteobacteria* on leaves suggests niche specialization for plant compartments. Location significantly shaped both community composition and fungal diversity on both plant parts. Notably, soil type influenced community composition but not diversity. Altitude was associated with increased fungal species diversity on leaves and flowers. Furthermore, significant effects of plant family identity emerged within a subset of seven families, impacting bacterial and fungal abundance, fungal Shannon diversity, and bacterial species richness, particularly on flowers.

**Conclusion:**

This study provides novel insights into the specific microbiome of wild flowering plants, highlighting adaptations to local environments and plant–microbe coevolution. The observed specificity indicates a potential role in plant health and resilience, which is crucial for predicting how microbiomes respond to changing environments, ultimately aiding in the conservation of natural ecosystems facing climate change pressures.

**Supplementary Information:**

The online version contains supplementary material available at 10.1186/s12866-024-03574-0.

## Background

Plants harbor diverse microbial communities on both leaves, responsible for photosynthesis and nutrient exchange, and flowers, which are essential for reproduction. Despite their crucial roles in plant health and function, our understanding of these microbial inhabitants, especially in natural settings, remains limited. Floral nectar enriches a dynamic microbiota of yeasts and bacteria crucial for plant health and reproduction [1]. Specific microbial diversity and networking was found to be reflected in seeds developed from flowers of alpine plants as well [2]. Previous studies comparing microbial communities in flowers and leaves, mainly in crops, have yielded conflicting results, highlighting the need for further exploration across diverse wildflower families in natural settings. For instance, some studies have reported lower microbial diversity in flowers compared to leaves in species like *Holcus lanatus*, *Trifolium pratense*, and *Achillea millefolium* [3, 4]. However, other studies have found either higher bacterial diversity on flowers [5] or no significant differences in bacterial richness between flowers and leaves [6].These contrasting findings highlight the need for further exploration. While previous research has often focused on bacterial communities in plant aerial parts, incorporating fungal communities is essential for a comprehensive understanding of plant–microbe interactions, particularly in leaves and flowers. Fungi play diverse roles in these tissues, for instance epiphytic fungi on leaf surfaces can modulate microclimatic conditions and offer protection against environmental stressors [7]. Additionally, flowers harbor distinct fungal communities crucial for both pollination and disease dynamics. Insect visitation significantly increases fungal richness [8], highlighting the dependence of floral fungi on insect-mediated dispersal. Moreover, many floral diseases are caused by fungal pathogens [9, 10], underscoring the importance of understanding fungal communities in flower health and reproduction. The amazing diversification within the plant kingdom can of course produce different pattern, but it is also important to understand general rules in microbial ecology of plants.

Alpine ecosystems are characterized by diverse plant communities and exhibit distinct variations along the altitudinal gradient. The “calcareous riddle” describe the enormous diversity of calciphilous species in alpine mountains [11]. Soil type, through its influence on soil microbial communities, indirectly shapes the microbiomes of aerial plant parts, including leaves and flowers. Recent research demonstrates a significant proportion of bacteria on leaves and flowers originate from the soil [12, 13], highlighting soil's role as a primary microbial source. Edaphic factors such as pH and nutrient availability influence soil microbiota composition, which serves as a reservoir for plant colonization [13]. Moreover, soil microbiomes can directly impact key plant traits like flowering time [14], indirectly influencing aerial plant part composition. While flowers actively shape their microbial communities through selective enrichment [12], the foundational influence of soil type on aerial plant microbiomes underscores the importance of understanding soil–plant-microbe interactions in shaping the composition and function of leaf and flower microbiomes. Environmental factors play a crucial role in shaping the composition and function of these aerial microbiomes. Evidence suggests that microbial communities in these aerial plant compartments can exhibit local adaptation to specific environmental conditions [15–17]. For example, a study by Gaube et al. [18] revealed that while plant species- and organ-specific bacterial communities associated with flowers and leaves did not differ significantly between distinct biogeographic regions, there was considerable variation within locations [18]. This suggests that local environmental factors may play a more dominant role in shaping aerial microbiomes than broad geographical patterns. Abiotic factors such as temperature [19], UV radiation [20], and wind [21] can also impact the assembly and structure of floral and leaf microbiomes. Temperature and UV radiation, which vary significantly with altitude, have been shown to influence both bacterial and fungal communities on leaves [22]. For instance, Yang et al. [22] found that bacterial diversity increased while fungal diversity decreased with elevation in the phyllosphere of *Kobresia pygmaea* on the Tibetan Plateau [22]. Additionally, Wang et al. [23] observed a monotonic decline in both bacterial and fungal diversity with increasing elevation [23]. These findings suggest that altitude, through its influence on abiotic factors, plays a key role in shaping the microbial communities not only in leaves but also in flowers. However, to our knowledge, no studies have explicitly investigated the combined effects of altitude and geographical location on the microbiomes of both leaves and flowers. In addition to abiotic factors, plant species identity, and consequently, plant family membership, plays a significant role in shaping the composition of its associated microbiome [24]. Closely related plant species harbour similar microbial communities compared to distant ones and this phenomenon termed “phylosymbiosis” [25]. This pattern is particularly evident in endophytic communities, highlighting the role of inheritance and vertical transmission [26]. Recent studies by Abdelfattah et al. [15, 27] further explore this concept, emphasizing the crucial role of microbial inheritance in shaping and transmitting plant microbiomes across generations [15, 27]. Furthermore, host genotype directly influences microbiome composition and abundance. Wagner et al. [28] demonstrated the influence of plant genotype on the leaf microbiome [28], while Bodenhausen et al. [29] identified specific mutations altering the phyllosphere microbiota [29]. This influence extends to fruits and seeds, where heritability plays a significant role [30–32]. Although physiological traits and host genotype effects might not always perfectly align with plant phylogeny [33], shared characteristics within families or orders suggest potential convergence in microbiome traits, regardless of evolutionary relationships.

**Fig. 1:**
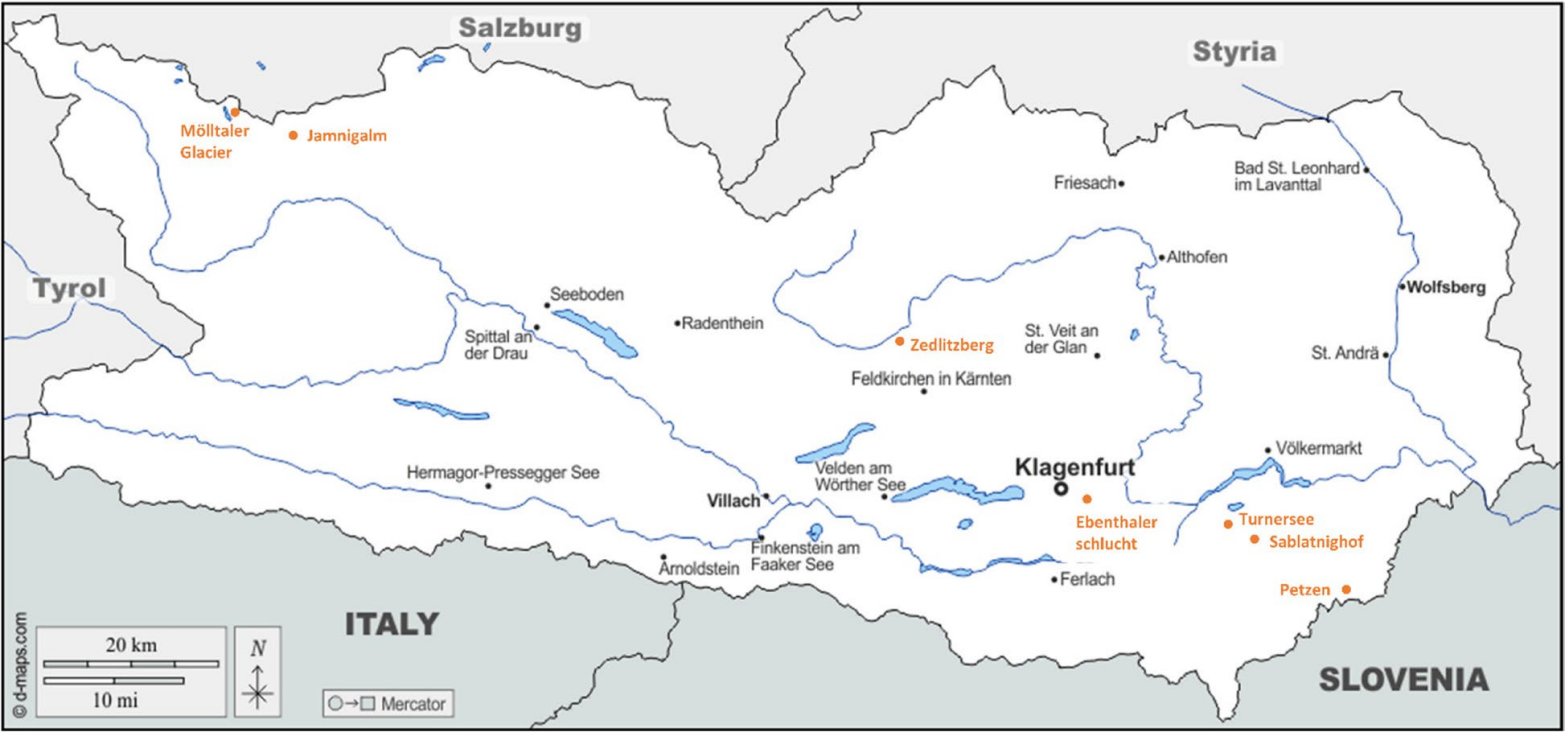
Sampling locations in Carinthia, Austria. Red dots indicate the seven locations where plant samples were collected. The exact coordinates of the sampling locations are provided in Supplementary file

This study aims to answer three key questions (Fig. 1), 1. Do flowers and leaves harbor distinct microbial communities in terms of diversity, abundance, and composition? 2. How do geographical location, altitude, and soil type influence microbial diversity, abundance, and composition of plant organs? 3. Does plant family identity affect the diversity, abundance, and composition of associated microbial communities? To address these knowledge gaps in our understanding of leaf and flower microbiomes, we focused our investigation on the floral and epiphytic (leaf) microbiomes of wild flowering plants in the Austrian Alps. We hypothesize that the unique environmental conditions of alpine habitats, characterized by its diverse plant communities, high altitudes, fluctuating temperatures, and distinct soil types may select for specialized microbial communities adapted to these challenging environments. We employed amplicon sequencing and qPCR to analyze the bacterial and fungal communities associated with both flowers and leaves.

## Material and methods

### Study area

We collected leaves and flowers of 50 flowering plants during 25–30 July 2021 at seven different locations (Ebenthalerschlucht, Turnersee, Petzen, Sablatnighof, Zedlitzberg, Mölltaler Glacier and Jamnigalm) in Carinthia, Austria (Fig. 1). The altitude of these sampling locations ranged from 450 – 2760 m. Based on geological maps of Carinthia, we classified the soil types into two broad categories: carbonate and silicate soils, which primarily reflect differences in the underlying geology and mineralogy. While this classification provides a general overview of soil types, it is important to note that it does not fully capture the complex variations in soil chemistry, such as pH and organic matter content, that can significantly influence microbial communities. Future studies incorporating detailed soil analyses would provide a more comprehensive understanding of the relationship between soil characteristics and plant microbiomes. While silicate soil can be assumed at Zedlitzberg and Mölltaler Glacier, the Petzen is part of to the eastern limestone Alps and therefore carbonate bedrock can be found here. Regarding the other sampling locations, both carbonate and silicate components can occur. While carbonate components are more likely to be found at the sampling locations Ebenthalerschlucht, Turnersee and Sablatnighof, based on the photographic documentation evaluation mainly carbonate bedrock is assumed at the sampling location Jamnigalm.

### Plant species

The 50 flowering plant species investigated belong to 22 families and include typical alpine flowers as for example *Gentiana bavariaca* or *Rhododendron ferrugineum* as well as well-known medicinal plants like *Arnica montana*. For each species, we collected one sample consisting of the entire flower and one sample of leaves from a single individual plant at each location, resulting in a total of 100 samples (50 flower and 50 leaf samples). We acknowledge that different floral organs may harbor distinct microbial communities. However, due to the constraints of this study, we focused on characterizing the overall flower microbiome. No technical replicates were included in this study. A list of all the plant species with information on family identity, sampling location, coordinates, date of sampling, altitude, soil type and sample condition can be found in the Supplementary Table 1. Samples were kept at 4 °C during transport to the laboratory, the stored at—70 °C. One gram of leaves or flowers was lyophilized for 48 h using the Labconco Freeze Dry System (Labconco, Kansas City, USA). After lyophilization the samples were stored at -20 °C until DNA extraction.

### Microbial DNA extraction and amplicon library construction

Lyophilized plant samples (100 mg) were homogenized in 2 ml tubes using FastPrep Instrument (MP Biomedicals, Illkirch, France) with different sized glass beads (0.25–0.5 mm and 2.85–3.48 mm) for 60 s at 5 m/s. The resulting lysate was transferred to Lysing Matrix E tubes with glass beads and DNA extraction was performed according to the FastDNA SPIN Kit for Soil (MP Biomedicals, Solon, OH, United States) instructions.

The 16S rRNA gene was amplified using primers 515f and 806r in conjunction with PNA clamps to block host plastid and mitochondrial DNA amplification. Each 10 μl PCR reaction contained 6.5 μl PCR-grade water, 0.1 μl each of 515f and 806r primers (10 μM), 0.15 μl each of mPNA and pPNA (50 μM), 2.0 μl Taq&Go 5X (MP Biomedicals, Illkirch, France), and 1 μl of 1:10 diluted template DNA. Negative controls used PCR-grade water instead of DNA. PCR conditions were: initial denaturation at 95 °C for 5 min, followed by 35 cycles of 95 °C for 30 s, 78 °C for 5 s, 55 °C for 30 s, and 72 °C for 60 s, with a final extension at 72 °C for 5 min. A second PCR using the first PCR product as template added barcodes to the amplified regions. The reaction mix (30 μl) contained 19.6 μl PCR-grade water, 1.2 μl each of Golay barcode forward and reverse primers (5 5 μM), 6.0 μl Taq&Go (MP Biomedicals, Illkirch, France), and 2 μl of the first PCR product. Cycling conditions were: initial denaturation at 95 °C for 5 min, followed by 15 cycles of 95 °C for 30 s, 53 °C for 30 s, and 72 °C for 30 s, with a final extension at 72 °C for 5 min. Amplified DNA was purified using the Wizard SV Gel and PCR Clean-Up System (Promega Corporation, Madison, USA). Purified libraries and a negative control were sequenced on an Illumina MiSeq at Eurofins Genomics Europe Sequencing GmbH (Konstanz, Germany).

The ITS1 region was amplified using primers ITS1f and ITS2rp. The PCR reaction (30 μl) contained 21.8 μl PCR-grade water, 0.6 μl each of ITS1f_XXXBC and ITS2rp_XXXBC primers (10 μM), 6.0 μl Taq&Go (MP Biomedicals, Illkirch, France), and 1 μl of undiluted template DNA. Negative controls used PCR-grade water instead of DNA. Triplicates were performed for each sample and visualized on agarose gels. Samples with failed amplification were excluded from further analysis. Amplified DNA was purified using the Wizard SV Gel and PCR Clean-Up System (Promega Corporation, Madison, USA) and pooled prior to Illumina amplicon sequencing at Eurofins Genomics (Konstanz, Germany).

### Quantitative PCR (qPCR)

Bacterial and fungal abundance were quantified using real-time PCR (qPCR) targeting the 16S rRNA gene for bacteria and ITS rDNA for fungi. A Rotor-Gene 6000 analyzer (Corbett Research, Sydney, Australia) was used with standard curves generated from known-copy-number *Pseudomonas* and *Penicillium* isolates. For bacterial analysis, the reaction mixture contained 5 μL of KAPA SYBR Green, 0.15 μL of each primer (mPNA and pPNA), 0.5 μL each of universal bacterial primers (unibacII 515f and 806r), 2.7 μL of PCR-grade water, and 1 μL of template DNA. Cycling conditions were: 95 °C for 5 min, followed by 40 cycles of 95 °C for 20 s, 78 °C for 5 s, 54 °C for 15 s, and 72 °C for 30 s. A final melt curve analysis was performed from 72 °C to 96 °C. Similarly, for fungal analysis the reaction mixture contained 5 μL of KAPA SYBR Green, 0.5 μL each of ITS1 and ITS2 primers, 1 μL of template DNA, and PCR-grade water. Cycling conditions were 95 °C for 3 min, followed by 40 cycles of 95 °C for 5 s, 58 °C for 35 s, and 72 °C for 5 s. A final melt curve (72 °C-95°C) confirmed fungal amplicon specificity. PCR-grade water was used as a negative control in both cases.

### Bioinformatics and statistical analysis

Raw sequences were demultiplexed using cutadapt [34] with strict quality control, discarding mismatches, indels, and untrimmed reads. QIIME2 [35] was used for further processing, employing DADA2 [36] to trim low-quality reads, remove chimeras, and create amplicon sequence variants (ASVs). ASV taxonomy was assigned using VSEARCH [37] against SILVA v138 [38] and UNITE V 8.3 [39] databases. Sequences from mitochondria, chloroplasts, and unclassified sources were filtered out. Reads were rarefied to even depths (7,000 for fungi; 1,700 reads/sample for bacteria) to account for unequal sequencing depth when calculating diversity indices. Cumulative Sum Scaling (CSS) [40] transformed bacterial and fungal data for community composition analyses. To assess the difference between leaves and flowers, Shannon diversity, species richness, and abundance were modeled using linear mixed-effects models (lme4 package) [41], with plant compartment (fixed) and sample condition (random) as factors. PERMANOVA (adonis2; vegan package) [42] was used to test for differences in bacterial and fungal communities based on plant compartment. *P*-values for diversity and abundance originated from model summaries, while community composition *p*-values were derived from PERMANOVA. *R*-squared values for diversity and abundance were calculated using r.squaredGLMM function MuMIn package [43]. Since the difference between flowers and leaves was found to be significant, separate analyses were conducted for flowers and leaves, with rarefaction depths adjusted based on sample variability. The fungal dataset was rarefied to an even depth of 7500 reads/sample for flowers, and 7000 reads/ sample for leaves. The bacterial dataset was rarefied to 7900 reads/sample for flowers and 1700 reads/sample for leaves. Shannon diversity, richness, and abundance were modeled using linear regression (stats package) [44] with sampling location and soil type as fixed effects and sample condition as random effect. Community composition was tested using PERMANOVA (adonis2).

To assess the impact of family identity on microbial community composition and diversity, a subgroup of 29 species representing seven different families (*Apiaceae, Asteraceae, Caryophyllaceae, Fabaceae, Lamiaceae, Ranuculaceae*, and *Rosaceae*) was used. Rarefaction depths were adjusted to 28,500 reads/sample for flowers and 1200 reads/sample for leaves for the fungal community and 7900 reads/sample for flowers and 2300 reads/sample for leaves for the bacterial community. These rarefaction depths were chosen based on the minimum number of reads observed in each respective dataset, ensuring the inclusion of all samples while maintaining a consistent sequencing depth across the dataset. Shannon diversity, species richness and abundance were modelled separately as a function of family identity as fixed effect and sample condition as random effect using ANOVA (car package) [45] was used to test for significance, with marginal *R*-squared values calculated using the MuMIn package. To assess the effect of altitude, Shannon diversity, richness, and abundance were modeled using linear regression with the function 'lm' from the stats package [44] with altitude as the predictor. Core microbiomes were identified using the "microbiome" package [46, 47] at each altitude level. Community composition was tested using PERMANOVA (adonis2). Bray–Curtis dissimilarities and principal coordinate analysis (PCoA) were used to visualize community dissimilarity (phyloseq package) [48]. Linear discriminant analysis Effect Size (LEfSe) [49] was used to identify taxa with significant differential abundance across groups, we used "microbiomeMarker" [50] package in R to perform LEfSe analysis. The run_lefse function was used to identify differentially abundant taxa between groups (flowers vs. leaves) at the genus level. Normalization was performed using centered log-ratio (CLR) transformation ("CSS" parameter). Only taxa with significant Kruskal–Wallis test results (*p*-value < 0.05) and an LDA score > 2 were considered for further interpretation.

## Results

### Evidence for Niche specialization in leaf and flower microbiomes

**Fig. 2:**
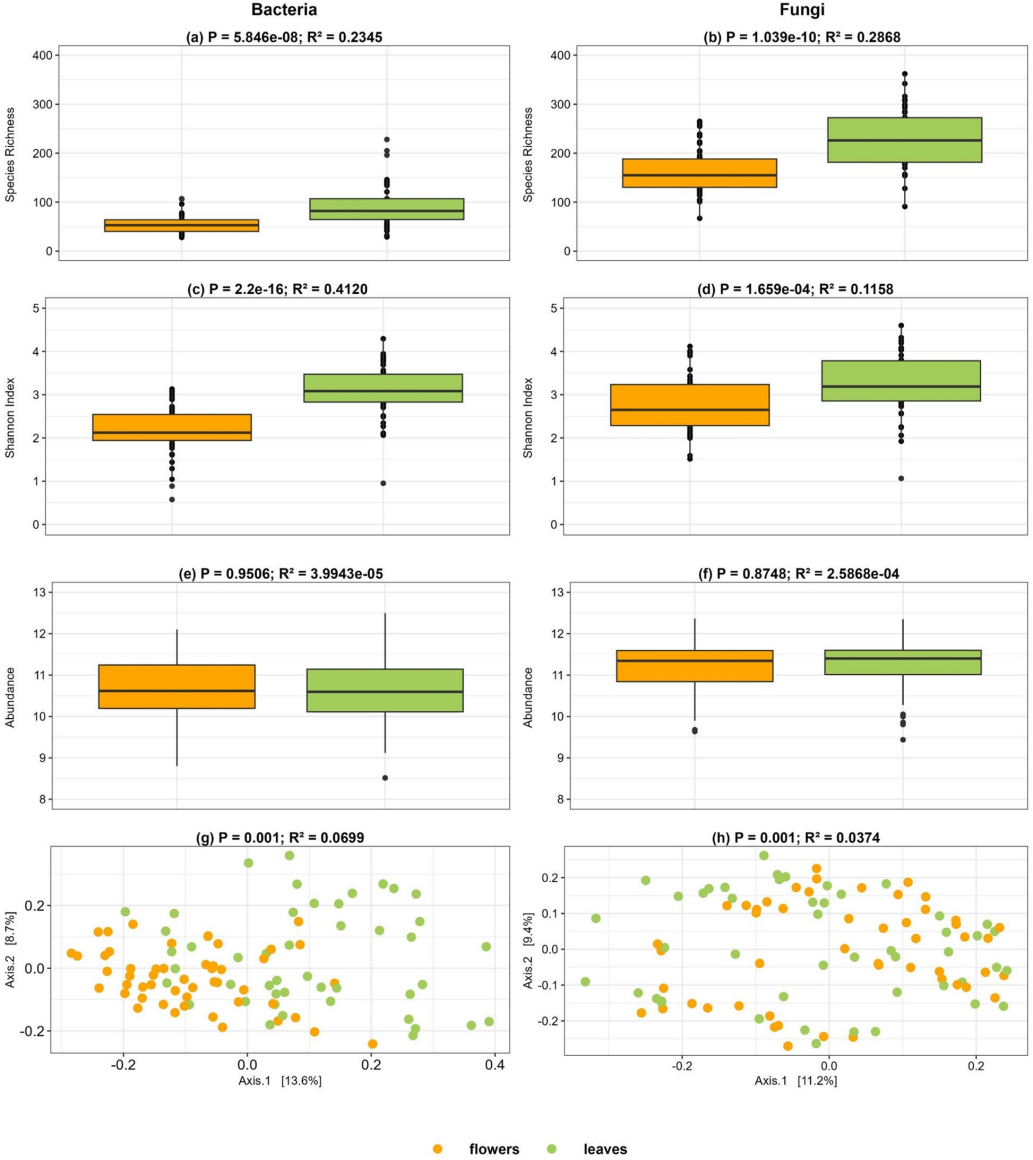
Box and Principal Coordinate Analysis (PCoA) plots illustrating the difference between flowers and leaves in terms of bacterial and fungal species richness (**a**, **b**), shannon index (**c**, **d**), and abundance (**e**, **f**), as well as the impact of the plant compartment on the microbial community composition (**g**, **h**) of the sampled flowers and leaves

**Fig. 3:**
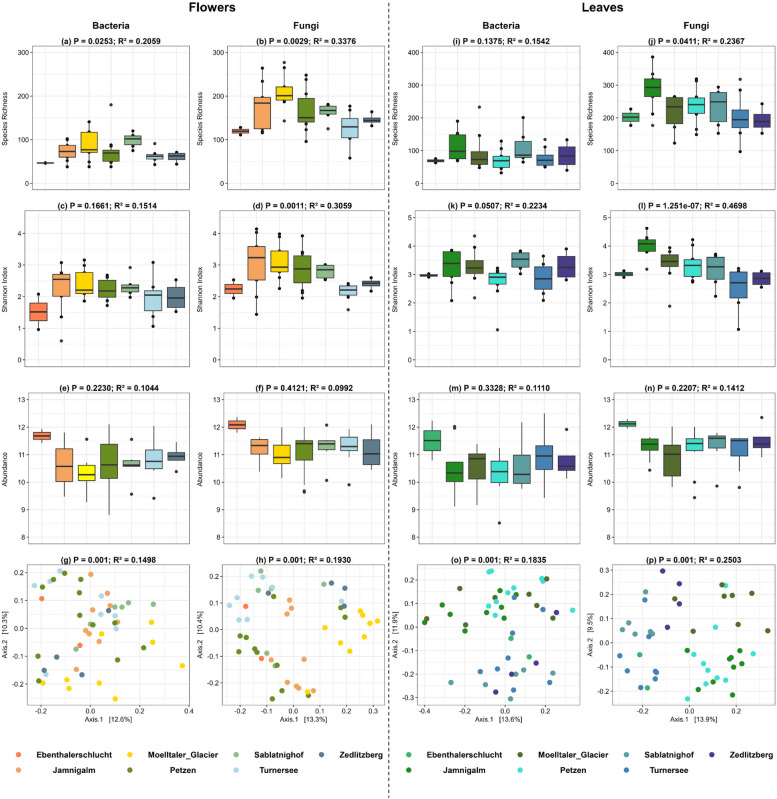
Bacterial and Fungal diversity, community composition in flowers and leaves at different locations. Box plots illustrate species richness (**a**, **b**, **i**, **j**), shannon index (**c**, **d**, **k**, **l**), and abundance (**e**, **f**, **m**, **n**) in both bacterial and fungal. PCoA plots (**g**, **h**, **o**, **p**) illustrate the bacterial and fungal community composition. Panels are organized to highlight the corresponding metrics for flowers and leaves communities in the specified compartments

Leaves exhibited significantly higher bacterial and fungal species richness compared to flowers (Fig. 2a, b. On average, leaves harboured 228 fungal and 91 bacterial ASVs, whereas flowers had an average of 163 fungal and 55 bacterial ASVs. Similarly, leaves showed significantly higher bacterial and fungal Shannon diversity compared to flowers (Fig. 2c and d). However, no significant differences were observed in the abundance of bacterial 16S or fungal ITS gene copies between leaves and flowers (Fig. 2e and f). On average, flowers contained approximately 1.7122 × 10^11^ bacterial and 3.3570 × 10^11^ fungal gene copies per gram, while leaves harboured an average of 2.5610 × 10^11^ bacterial and 3.5802 × 10^11^ fungal gene copies per gram. Analysis of the bacterial and fungal community composition using Bray–Curtis dissimilarity revealed significant differences between leaves and flowers for both bacteria and fungi (Fig. 3g and h; Supplementary Table 2a).

The result of linear discriminant analysis (LEfSe) revealed significant differences in the abundance of several microbial genera between flowers and leaves (Supplementary Fig. 1a, b). Notably, bacterial genera *Pseudomonas* and *Pantoea* were enriched on flowers, while *Sphingomonas* and *Methylobacterium-Methylorubrum* were more abundant on leaves. Similarly, the fungal genera *Metschnikowia* and an *unidentified Sclerotiniaceae* were significantly associated with flowers, while *Ramularia* and an *unidentified Mycosphaerellaceae* exhibited higher abundance on leaves. These significant differences in microbial community composition between flowers and leaves underscore the presence of specialized niches within distinct plant compartments, justifying our decision to analyze them separately.

### The effect of geographical location on bacterial and fungal communities

In flowers, sampling location significantly influenced bacterial and fungal species richness (Fig. 3a, b). Although bacterial Shannon diversity was not significantly affected fungal Shannon diversity was influenced by sampling location (Fig. 3c, d). Sampling location did not significantly affect the abundance of bacterial and fungal gene copy numbers (Fig. 3e, f). Bacterial and fungal community composition was significantly influenced by sampling location (Fig. 3g, h). In leaves, sampling location had a significant effect on fungal, but not the bacterial, species richness and Shannon diversity (Fig. 3-l). Similar to flowers, sampling location did not significantly affect bacterial and fungal abundance on leaves (Fig. 3m, n). Notably, sampling location had a significant influence on bacterial and fungal community composition (Fig. 3o, p; Supplementary Table 2b).

### The effect of soil type on bacterial and fungal communities

**Fig. 4:**
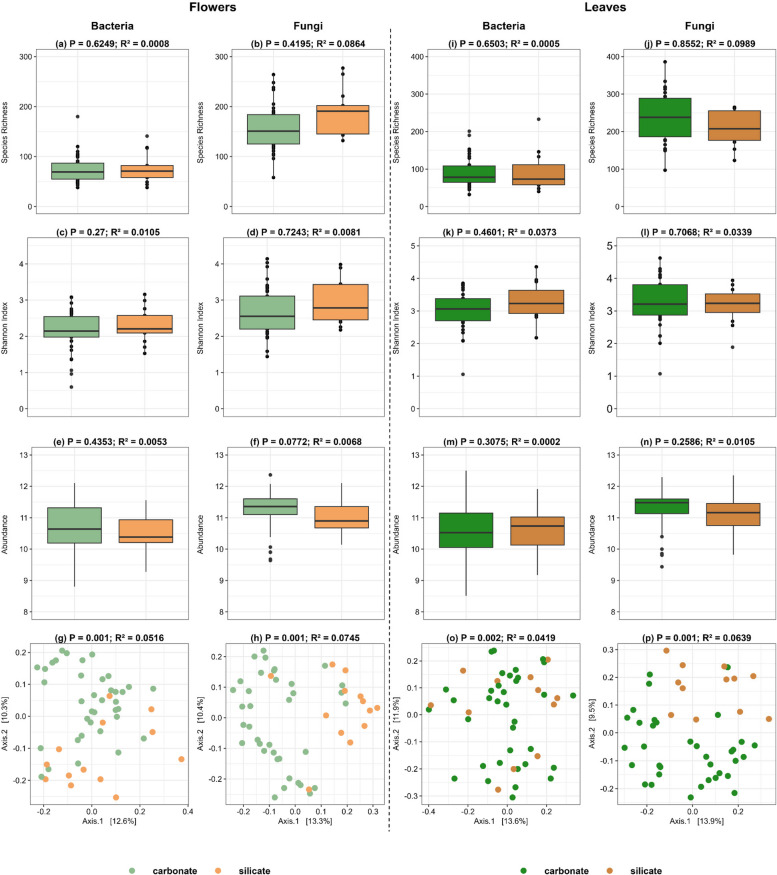
Bacterial and Fungal diversity, community composition in flowers and leaves for different soil types. Box plots illustrate species richness (**a**, **b**, **i**, **j**), shannon index (**c**, **d**, **k**, **l**), and abundance (**e**, **f**, **m**, **n**) in both bacterial and fungal. PCoA plots (**g**, **h**, **o**, **p**) illustrate the bacterial and fungal community composition. Panels are organized to highlight the corresponding metrics for flowers and leaves communities in the specified compartments

Soil type did not have a significant effect on fungal or bacterial species richness, Shannon diversity, and abundance in both flowers and leaves (Fig. 4a-f, i-p). In contrast, soil type had a significant effect on bacterial and fungal community composition in both flowers and leaves (Fig. 4g-p; Supplementary Table 2b).

### The effect of altitude on bacterial and fungal communities

**Fig. 5:**
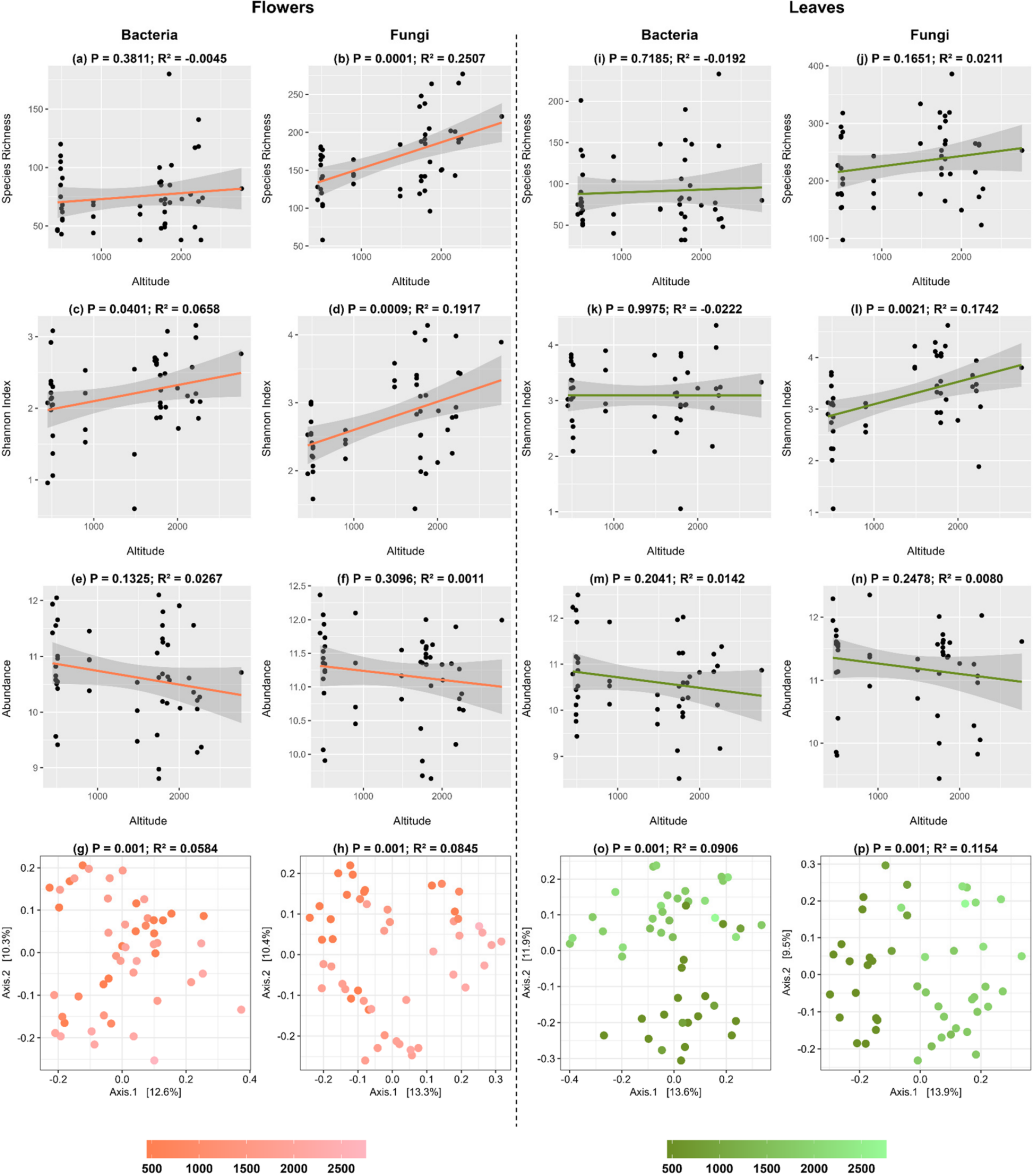
Bacterial and Fungal linear regressions and PCoA plots in flowers and leaves at different altitudes. Species richness (**a**, **b**, **i**, **j**), shannon index (**c**, **d**, **k**, **l**), and abundance (**e**, **f**, **m**, **n**) were all modelled separately using a linear regression with the function 'lm' (stats package) with statistical relevant values being extracted from the linear regression. PCoA plots (**g**, **h**, **o**, **p**) illustrate the bacterial and fungal community composition. Panels are organized to highlight the corresponding metrics for flowers and leaves communities in the specified compartments

**Fig. 6:**
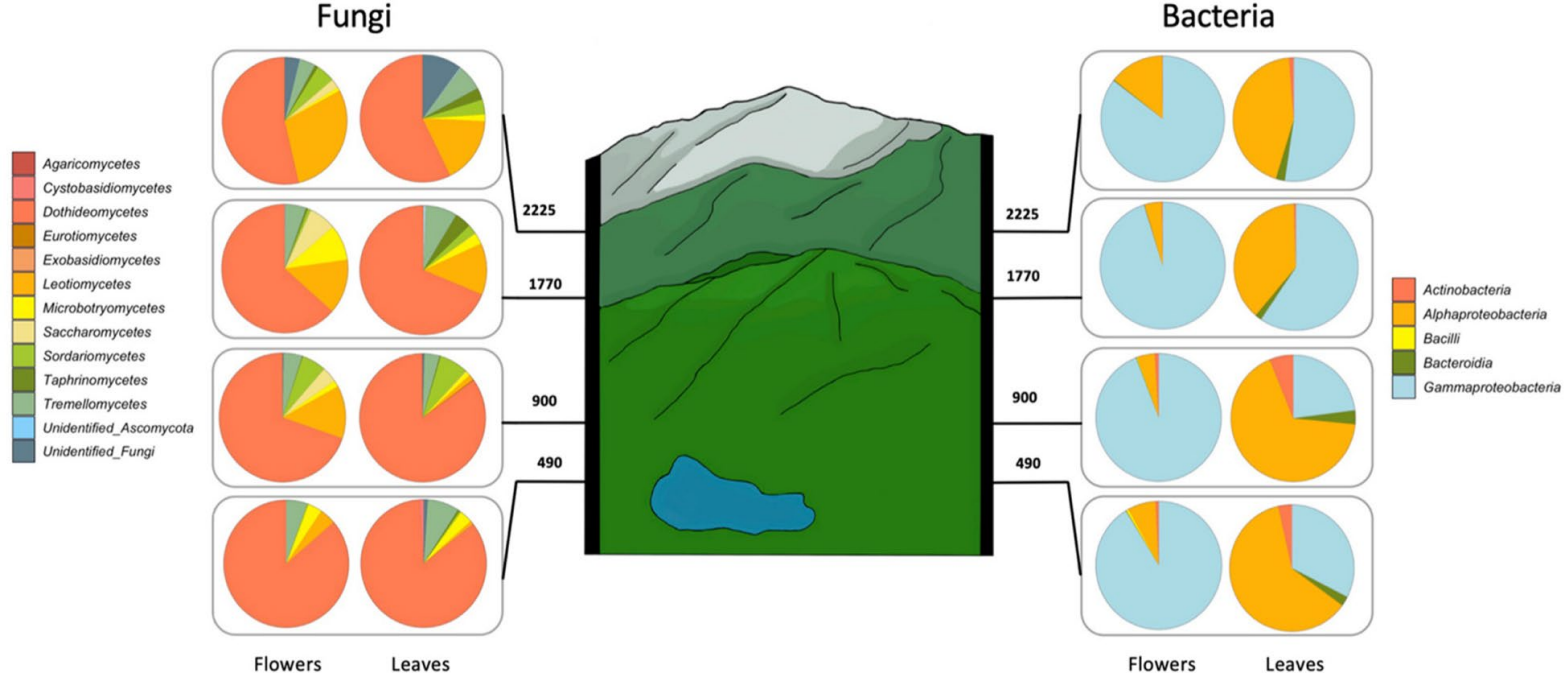
Bacterial and fungal core microbiome assessed for four different altitude levels on flowers as well as on leaves. Core microbiome was defined as classes present in at least 70% of the flowers or leaves sampled and determined using the 'core' function of the microbiome package

Altitude has significant effect on the flowers’ fungal but not bacterial species richness (Fig. 5a, b) and a significant effect on both fungal and bacterial Shannon diversity (Fig. 5c, d). Fungal species richness increased from approximately 130 ASVs at the lowest altitude (490 above sea level) to 215 ASVs at the highest altitude (2225 above sea level). Altitude did not have a significant effect on the abundance of bacterial and fungal gene copy numbers, but linear regressions showed a slight decline in bacterial and fungal abundance with increasing altitude (Fig. 5e, f). The influence of altitude on bacterial and fungal community composition was significant (Fig. 5g, h). In the case of leaves, a significant increase with altitude was only observed for fungal Shannon diversity with increasing altitude, and not on fungal and bacterial species richness, nor bacterial Shannon diversity (Fig. 5i-l). Although altitude did not have a significant effect on bacterial and fungal abundance in leaves, a slight decrease in abundance was noticeable with increasing altitude (Fig. 5 m, n). PERMANOVA analysis indicated that altitude had a significant effect on bacterial and fungal community composition (Fig. 5o, p; Supplementary Table 2b). As altitude was found to have a significant influence on bacterial and fungal communities, we examined the bacterial and fungal core microbiome (defined as classes present in at least 70% of the sampled flowers or leaves) across four different altitude levels (Fig. 6). A decrease in the relative abundance of *Dothideomycetes* was observed with increasing altitude, while the relative abundance of the class *Leotiomycetes* showed an increase on both flowers and leaves as altitude increased. On the bacterial front, the core microbiome consisted of only five classes. *Gammaproteobacteria* emerged as the dominant class on flowers, while on leaves, the class *Alphaproteobacteria* prevailed. Furthermore, the relative abundance of *Alphaproteobacteria* appeared to be higher at lower altitudes on leaves.

### The effect of plant family identity on microbial diversity, abundance and community composition

**Fig. 7:**
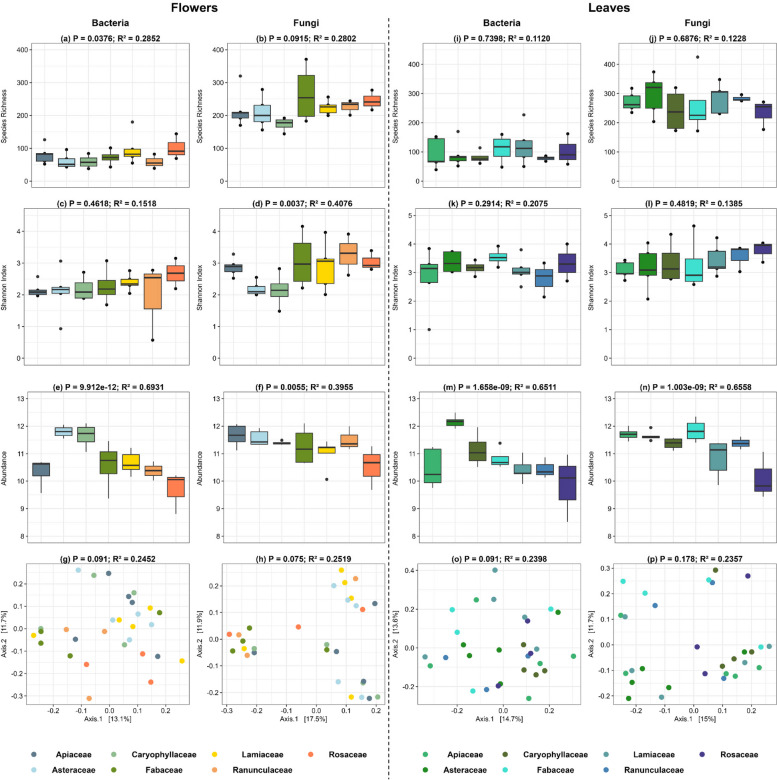
Bacterial and Fungal diversity, community composition in flowers and leaves for the different plant family identity. Box plots illustrate species richness (**a**, **b**, **i**, **j**), shannon index (**c**, **d**, **k**, **l**), and abundance (**e**, **f**, **m**, **n**) in both bacterial and fungal. PCoA plots (**g**, **h**, **o**, **p**) illustrate the bacterial and fungal community composition. Panels are organized to highlight the corresponding metrics for flowers and leaves communities in the specified compartments

To examine the influence of plant family identity on microbial diversity, abundance, and community composition, a subgroup consisting of 29 flowering plants from seven different families (*Apiaceae, Asteraceae, Caryophyllaceae, Fabaceae, Lamiaceae, Ranunculaceae, Rosaceae*) was formed. Each family included at least three different species. In the case of flowers, family identity was found to have a significant influence on bacterial species richness and fungal Shannon diversity (Fig. 7a, d). However, no significant effect was observed on bacterial Shannon diversity or fungal species richness (Fig. 7c, b). The influence of family identity on bacterial and fungal abundance was assessed using qPCR measurements. The results showed that family identity had a significant effect on bacterial and fungal abundance (Fig. 7e, f). Regarding bacterial and fungal community composition, no significant influence of family identity was observed (Fig. 7 g, h). In the case of leaves, no significant influence of family identity on bacterial species richness and Shannon diversity was observed (Fig. 7i, k). Similarly, no significant effect of family identity was found on fungal species richness and Shannon diversity (Fig. 7j and l). As for leaves, family identity exhibited a significant influence on the abundance of bacterial and fungal gene copy numbers (Fig. 7 m, n). In terms of bacterial and fungal community composition, family identity did not show a significant influence (Fig. 7o, p; Supplementary Table 2b).

## Discussion

In this study, we analysed the microbial communities associated with flowers and leaves of 50 wild flowering plants from different plant families, and found statistically significant impact of biotic and abiotic factors on their structure. We observed distinct microbial compositions between flowers and leaves, with leaves harbouring higher microbial diversity than flowers. We showed that sampling location had significant effect on diversity and community composition. Additionally, soil type had a significant impact on microbial community composition but not on diversity. Altitude showed a significant correlation with increased fungal species richness on flowers and elevated diversity on both leaves and flowers. Family identity effects were explored in a subset of seven plant families, revealing notable influences.

### Evidence for Niche Specialization in Leaf and Flower Microbiomes

Our results showed that despite of the variations in sampling location, altitude, soil type, and plant species, leaves harboured higher bacterial and fungal diversity compared to flowers. The disparity in microbial diversity between leaves and flowers could be attributed to the shorter lifespan of flowers compared to leaves coupled with the nutrient-rich nature of flowers. Flowers, once open, represent a new, resource-rich niche, where primary colonizers are expected to be R strategists (fast growing microorganisms) due to the limited competition and surplus of resources. The dominance of these faster-growing species together with the short lifespan of flowers, may limit the establishment of diverse microbial communities. In fact, genera such as *Pseudomonas* and *Pantoea*, and *Metschnikowia* were detected at significantly higher abundant on flowers than on leaves; all of which are recognized as r-strategist genera [51–54]. The presence of floral volatiles, such as terpenes with known antibacterial and antifungal properties [55], could contribute to the lower microbial diversity observed in flowers. These compounds may act as selective agents, limiting successful colonization to microbial taxa capable of tolerating or metabolizing them. In contrast, leaves provides a more stable and prolonged habitat for new microbial colonization. The higher fungal diversity observed on leaves might be related to the fluctuating nature of leaf senescence and associated changes in plant defense mechanisms. As leaves age, their antioxidant defense systems decline, potentially making them more susceptible to fungal colonization [56]. The enrichment of *Ramularia* on leaves, a genus known to include leaf pathogens favored by prolonged wetness [57], aligns with this pattern. Our study revealed distinct microbial composition in leaves and flowers, with significant differences observed in both bacterial and fungal community composition. These differences may be attributed to the unique characteristics of leaves and flowers, including their varying nutritional content, metabolite profiles, and susceptibility to pathogen attack. For instance, flowers often accumulate higher concentrations of secondary metabolites like flavonoids compared to leaves [58], which could potentially influence the microbial composition. Additionally, the susceptibility of flowers to pathogen attack due to their rich nutrient and moisture content, and high frequency of insect visitors [59], may also contribute to the observed differences in microbial composition. Furthermore, the enrichment of *Metschnikowia* in flowers aligns with its known association with floral nectar and its potential role in pollination and plant–insect interactions [60]. *Metschnikowia* species have even been shown to modify nectar chemistry and volatile profiles, potentially influencing pollinator visitation rates [61], underscoring the close relationship between floral microbiomes and plant reproduction.

### The effect of geographical location on bacterial and fungal communities

Our study revealed distinct responses of fungal and bacterial communities to geographical location. Fungal diversity in both flowers and leaves, and bacterial species richness in flowers, were significantly influenced by location. Bacterial and fungal abundance remained unaffected, while community composition varied across locations. This highlights the potential sensitivity of fungal communities to environmental variations across the landscape. Consistent with findings by Coleine et al. [62], fungal communities are highly responsive to fluctuations in temperature, humidity, and microclimates. These variations could explain the observed location-specific diversity patterns [62]. For instance, thermophilic fungi at warmer locations or moisture-tolerant fungi in areas with higher humidity might shape unique communities across the landscapes. Additionally, Xiao et al. [63] suggest that temperature fluctuations can enhance stochasticity within microbial communities, potentially contributing to the observed difference in fungal communities [63]. While bacterial species richness in flowers responded to location, overall diversity patterns remained less pronounced, possibly due to their superior dispersal mechanisms allowing them to colonize diverse locations and potentially exhibit less location-specific diversity patterns [64]. The absence of detailed measurements of specific environmental factors like temperature and soil pH limits conclusive attribution of diversity patterns to specific drivers. The observed abundance pattern suggests that changes in diversity may not necessarily translate directly to changes in abundance. This observation aligns with the regional species pool theory, which posits that local communities are subsets of a larger regional pool, shaped by environmental filters [65]. In our study, the relatively limited environmental heterogeneity within our Carinthia sampling area compared to the broader region might have restricted the influence of location on bacterial diversity, potentially explaining the lack of significant abundance changes.

### The effect of soil type on bacterial and fungal communities

Although soil type (carbonate vs. silicate) did not have an effect on fungal and bacterial diversity and abundance, it significantly influence bacterial and fungal community composition. This observation suggests that soil type exerts a selective pressure on the microbial communities inhabiting above-ground plant parts. Recent research has demonstrated that soil microbiomes can significantly influence the composition of aerial plant microbiomes, suggesting that variations in soil type may indirectly shape the microbial communities on leaves and flowers through plant-mediated assembly processes [66]. Soil physicochemical properties have a substantial effect on the soil microbiome, which can subsequently influence phyllosphere microbiomes [67, 68]. One potential factor might be considered is phosphorus (P) availability, known to be crucial for both plants and microbes [69]. Carbonate and silicate soils have contrasting P profiles: high pH and low P in carbonate vs. neutral pH and moderate P in silicate [70]. These contrasting P profiles could directly influence the establishment and growth of specific microbial taxa on leaves and flowers, contributing to the observed compositional differences. While P-solubilizing microorganisms are primarily associated with the rhizosphere, the ability to solubilize phosphates has also been reported in phyllosphere microbes [68, 71]. Further research directly linking soil type differences to specific P-related effects on observed microbial composition is needed. Furthermore, studies like Sun et al. [72] demonstrated how organic matter type could influence soil fungal communities [72]. Similarly, we hypothesize that distinct plant communities associated with carbonate and silicate soils might contribute varying organic matter compositions, indirectly affecting microbial communities on leaves and flowers through resource availability and plant–microbe interactions. While our study relied on geological maps for soil type classification, future investigations directly analyzing soil properties could provide deeper insights into the influence of soil type on these microbial communities. These findings highlight the importance of considering the broader ecological context, including soil characteristics.

### The effect of altitude on bacterial and fungal communities

Our study revealed distinct responses for bacterial and fungal communities in flowers to the increasing altitude. While fungal diversity significantly increased with altitude, bacterial diversity was not affected. However, both communities displayed altered community composition and a slight decline in abundance across the altitudinal gradient. Lower temperatures and decreased oxygen availability at higher elevations might favour specific psychrophilic and hypoxia-adapted fungal communities [73] potentially leading to increased fungal richness and diversity. While temperature and oxygen availability are key factors, other abiotic conditions that change with altitude, such as UV radiation and wind exposure, could also contribute to the observed shift in fungal communities [20, 21]. While bacterial species richness remained stable, we observed shifts in community composition and a slight decline in abundance. Lindow & Brandl [74] suggest carbon-source availability as a major constraint for bacterial growth in the phyllosphere [74]. The observed decline in abundance for both fungi and bacteria could be attributed to combined effects of temperature, UV radiation [75], and potential resource limitations at higher altitudes [74]. Interestingly, only fungal Shannon diversity increased in leaves with altitude. This pattern aligns with the mechanisms mentioned for flowers, suggesting similar selection pressures and niche availability favouring specific fungal groups. Resource limitations at higher altitudes, particularly nitrogen availability [76], could explain why overall fungal and bacterial richness remained relatively stable despite the observed increase in fungal Shannon diversity. This suggests that while the evenness of fungal species distribution may increase with altitude, the total number of species present may not change significantly. The observed changes in community composition for both fungi and bacteria in leaves aligns with the findings by Sundqvist et al. [77] who demonstrated that altitudinal gradients significantly impact soil bacterial composition through associated changes in temperature and precipitation [77]. As leaves directly interact with this abiotic environment, similar selection pressures favouring specific adaptations to these changing conditions might explain the observed shifts in leaf bacterial communities.

Our analysis of the fungal core microbiome defined as classes present in at least 70% of sampled flowers and leaves across altitudes reveals distinct patterns (Fig. 6). Consistent with the temperature selection hypothesis, we observed a decline in *Dothideomycetes* and an increase in *Leotiomycetes* observed on both flowers and leaves. *Dothideomycetes* are often associated with warmer climates, limits their suitability at higher elevations, while *Leotiomycetes*, particularly the *Helotiales* order, harbor psychrophilic fungi found in alpine glaciers [78]. While temperature plays a crucial role, other factors might influence the core microbiome. Plant nitrogen availability linked to *Dothideomycete* presence [79], could be influenced by altitude. Additionally, *Dothideomycetes* are known for their tolerance to harsh conditions [80], suggesting potential adaptations beyond just temperature.

The bacterial core microbiota also displays notable patterns (Fig. 6). The dominance of *Gammaproteobacteria* on flowers and *Alphaproteobacteria* on leaves suggests niche specialization based on plant organ function. *Gammaproteobacteria*, known for their roles in plant growth promotion and pathogen protection [81] align with their prevalence in the flower microenvironment that is crucial for reproduction and seed development. *Alphaproteobacteria*, with their diverse metabolic capabilities [82], efficiently utilize and cycle nutrients on leaves. Their ability to maintain activity under environmental fluctuations is potentially advantageous in the leaf environment [83]. Interestingly, both bacterial classes exhibited contrasting trends with altitude. *Alphaproteobacteria* decline on leaves, potentially due to their lower tolerance for colder temperatures at higher altitudes [84], putting them at a disadvantage compared to psychrophilic *Gammaproteobacteria* [85], whose increased abundance aligns with this potential advantage. Additionally, *Alphaproteobacteria* might be sensitive to nitrogen limitations known to occur at higher altitudes [76], further contribute to their decline.

### The effect of plant family identity on microbial diversity, abundance and community composition

Our study observed a significant effect of family identity on bacterial species richness and fungal Shannon diversity on flowers, while no significant effect was observed on fungal species richness. In contrast, leaves only showed significant effect in bacterial and fungal abundance, but no changes in diversity and community composition. This aligns with previous findings by Redford et al. [86] and Knief et al. [87], suggesting that different plant families create distinct niches that favour specific microbial taxa [86, 87]. The observed diversity on flowers could be attributed to floral specificity where plant families likely possesses unique phytochemical profiles and nutrient content in their exudates [88, 89]. This aligns with studies demonstrating how host genotype shapes exudate profiles [90, 91], suggesting a link between family identity, exudate composition, and microbial communities. Our findings suggest that, lowest bacterial abundance in *Rosaceae* coincides with their known richness in hydrolysable tannins with antimicrobial and antifungal properties [92, 93]. The presence of these tannins in *Rosaceae* exudates could be suppressing microbial growth, contributing to their lower abundance compared to other families. Additionally, the presence of cyanogenic glycosides [94] might create a less favourable environment for some bacterial taxa [95], contributing to lower abundance. The higher abundance of bacterial and fungal communities observed on Asteraceae leaves and flowers correspond with their phytochemical richness, particularly in phenolic compounds and terpenoids [96, 97]. These secondary metabolites likely act as both selective agents and attractants, shaping the microbial community composition [98, 99]. The presence of essential oils, rich in volatile compounds like β-caryophyllene and myrcene [100], could further contribute to this selectivity, favoring microbes that can tolerate or even utilize these phytochemicals. Additionally, the antifungal properties of certain phytochemicals could promote beneficial fungal growth [101], contributing to the observed fungal abundance. Further studies characterizing specific phytochemical profiles and their antimicrobial properties across families are crucial to confirm this mechanism. In contrast to flowers, leaves exhibited lack of diversity and community composition despite the change in microbial abundance with family identity. This could be due to the higher baseline diversity and stronger environmental pressures experienced by leaves [28] might mask subtle diversity effects driven by family identity. This observation is consistent with the concept that host genotype effects and physiological traits may not always perfectly correlate with plant phylogeny [33]. Thus, while family identity influences microbial abundance, its impact on community composition might be less pronounced in leaves compared to other studies [25, 27], potentially due to the overriding influence of environmental factors and the inherent complexity of leaf microbiomes. Our findings also agree with Junker et al. [102], suggesting that microbial communities might be more organ-specific than species-specific, which is further supported by the stronger effect of family identity on flowers compared to leaves in our study [102]. While family-level analysis provides valuable insights, it is crucial to acknowledge phenotypic variations within species [103] and genotype-specific effects on microbial communities [104] can exist within families. Therefore, investigating species-level data within each family could provide a more clear understanding. Additionally, the regional species pool could also indirectly influence observed patterns [105, 106]. Differences in pollen and nectar availability across habitats could contribute to the observed abundance patterns, as suggested by Russell et al. [107].

## Conclusion

This study provides new ground in understanding the microbiome of wild flowering plants, highlighting distinct microbial communities associated with leaves and flowers. We observed higher microbial diversity and richness on leaves compared to flowers, suggesting niche specialization and adaptation to the unique challenges of each plant compartment. Furthermore, our findings reveal the significant influence of environmental factors such as location, soil type, altitude, and plant family identity significantly shaped microbial diversity, abundance, and composition, suggesting local adaptations and potential coevolutionary dynamics. While our study offers valuable insights, we acknowledge the limitations imposed by the relatively small sample size and the lack of detailed soil analyses. Future studies with larger sample sizes and more comprehensive environmental characterization will be crucial to further elucidate the complex interactions between wild flowering plants and their microbiomes.Understanding how these factors interact with the plant microbiome is critical for predicting responses to environmental changes particularly in the face of climate change pressures. This knowledge is crucial for predicting how plant microbiomes, both on leaves and flowers, will respond to environmental changes, ultimately aiding in the conservation of natural ecosystems and ensuring their sustainability.

## Supplementary Information

Supplementary Table 1: Details the characteristics of the investigated flowering plant samples. Supplementary Table 2a: Provides statistical comparisons (*p*-values and *R*-squared values) of microbial diversity, abundance, and community composition between flowers and leaves. Supplementary Table 2b: Presents statistical comparisons (*p*-values and *R*-squared values) for the effects of geographical location, soil type, altitude, and plant family on microbial diversity, abundance, and community composition. Supplementary Fig. 1: Visualizes differential microbial abundance between leaves and flowers using Linear Discriminant Analysis Effect Size (LEfSe) plots. Supplementary Fig. 2: Conceptual framework illustrating the factors investigated in this study.

## Data Availability

The datasets supporting the conclusions of this article are available in the Sequence Read Archive of NCBI. The raw sequence data 16S rRNA gene and ITS region are available under the BioProject ID PRJNA1081679.

## Abbreviations

ASV: Amplicon Sequence Variant
PCR: Polymerase Chain Reaction
DNA: Deoxyribonucleic Acid
rRNA: Ribosomal Ribonucleic Acid
