# Supplementary material for: Unravelling the microbiome of wild flowering plants: a comparative study of leaves and flowers in alpine ecosystems
Source: BMC Microbiol. 2024 Oct 19;24:417. doi: 10.1186/s12866-024-03574-0 (PMC11490174; doi:10.1186/s12866-024-03574-0)
Supplement: Supplementary file 1 — Supplementary Table 1: Details the characteristics of the investigated flowering plant samples. Supplementary Table 2a: Provides statistical comparisons (p-values and R-squared values) of microbial diversity, abundance, and community composition between flowers and leaves. Supplementary Table 2b: Presents statistical comparisons (p-values and R-squared values) for the effects of geographical location, soil type, altitude, and plant family on microbial diversity, abundance, and community composition. Supplementary Fig. 1: Visualizes differential microbial abundance between leaves and flowers using Linear Discriminant Analysis Effect Size (LEfSe) plots. Supplementary Fig. 2: Conceptual framework illustrating the factors investigated in this study. [file 12866_2024_3574_MOESM1_ESM.docx]

**Supplementary Table 1: Information of the samples on the flowering plants investigated**

| **ID** | **Compartment** | **Species** | **Family** | **Location** | **GPS** | **Coordinates** | **Date** | **ApproximateAltitude** | **Altitude** | **Soil_Type** |
| --- | --- | --- | --- | --- | --- | --- | --- | --- | --- | --- |
| 1f | flowers | Achillea_millefolium | Asteraceae | Sablatnighof | 242 | 46.571; 14.602 | 28.07.2021; 08:59:41 | 490 | 490 | carbonate |
| 1l | leaves | Achillea_millefolium | Asteraceae | Sablatnighof | 242 | 46.571; 14.602 | 28.07.2021; 08:59:41 | 490 | 490 | carbonate |
| 2f | flowers | Acinos_alpinus | Lamiaceae | Petzen | 252 | 47.008; 13.100 | 30.07.2021; 12:20:43 | 1770 | 1850 | carbonate |
| 2l | leaves | Acinos_alpinus | Lamiaceae | Petzen | 252 | 47.008; 13.100 | 30.07.2021; 12:20:43 | 1770 | 1850 | carbonate |
| 3f | flowers | Acinos_alpinus_ | Lamiaceae | Jamnigalm | 239 | 46.511; 14.769 | 27.07.2021; 11:41:48 | 1770 | 1860 | carbonate |
| 3l | leaves | Acinos_alpinus_ | Lamiaceae | Jamnigalm | 239 | 46.511; 14.769 | 27.07.2021; 11:41:48 | 1770 | 1860 | carbonate |
| 4f | flowers | Aconitum_lycoctonum | Ranunculaceae | Jamnigalm | 254 | 47.002; 13.119 | 30.07.2021; - | 1770 | 1486 | carbonate |
| 4l | leaves | Aconitum_lycoctonum | Ranunculaceae | Jamnigalm | 254 | 47.002; 13.119 | 30.07.2021; - | 1770 | 1486 | carbonate |
| 5f | flowers | Alchemilla_vulgaris | Rosaceae | Petzen | 237 | 46.515; 14.772 | 27.07.2021; 10:24:08 | 1770 | 1750 | carbonate |
| 5l | leaves | Alchemilla_vulgaris | Rosaceae | Petzen | 237 | 46.515; 14.772 | 27.07.2021; 10:24:08 | 1770 | 1750 | carbonate |
| 6f | flowers | Armeria_alpina | Plumbaginaceae | Petzen | 241 | 46.505; 14.766 | 27.07.2021; 15:12:36 | 2225 | 2010 | carbonate |
| 7f | flowers | Armeria_alpina_ | Plumbaginaceae | Moelltaler_Glacier | 244 | 47.015; 13.016 | 29.07.2021; 10:33:23 | 2225 | 2120 | silicate |
| 8f | flowers | Arnica_montana | Asteraceae | Jamnigalm | 251 | 47.006; 13.109 | 30.07.2021; 11:07:33 | 1770 | 1800 | carbonate |
| 8l | leaves | Arnica_montana | Asteraceae | Jamnigalm | 251 | 47.006; 13.109 | 30.07.2021; 11:07:33 | 1770 | 1800 | carbonate |
| 9f | flowers | Astrantia_major | Apiaceae | Petzen | 238 | 46.513; 14.771 | 27.07.2021; 11:03:41 | 1770 | 1795 | carbonate |
| 9l | leaves | Astrantia_major | Apiaceae | Petzen | 238 | 46.513; 14.771 | 27.07.2021; 11:03:41 | 1770 | 1795 | carbonate |
| 10f | flowers | Betonica_officinalis | Lamiaceae | Sablatnighof | 242 | 46.571; 14.602 | 28.07.2021; 08:59:41 | 490 | 490 | carbonate |
| 10l | leaves | Betonica_officinalis | Lamiaceae | Sablatnighof | 242 | 46.571; 14.602 | 28.07.2021; 08:59:41 | 490 | 490 | carbonate |
| 11f | flowers | Campanula_barbata | Campanulaceae | Jamnigalm | 251 | 47.006; 13.109 | 30.07.2021; 11:07:33 | 1770 | 1800 | carbonate |
| 11l | leaves | Campanula_barbata | Campanulaceae | Jamnigalm | 251 | 47.006; 13.109 | 30.07.2021; 11:07:33 | 1770 | 1800 | carbonate |
| 12f | flowers | Centaurea_nigrescens | Asteraceae | Ebenthalerschlucht | 233 | 46.598; 14.355 | 25.07.2021; - | 490 | 450 | carbonate |
| 12l | leaves | Centaurea_nigrescens | Asteraceae | Ebenthalerschlucht | 233 | 46.598; 14.355 | 25.07.2021; - | 490 | 450 | carbonate |
| 13f | flowers | Daucus_carota | Apiaceae | Sablatnighof | 242 | 46.571; 14.602 | 28.07.2021; 08:59:41 | 490 | 490 | carbonate |
| 13l | leaves | Daucus_carota | Apiaceae | Sablatnighof | 242 | 46.571; 14.602 | 28.07.2021; 08:59:41 | 490 | 490 | carbonate |
| 14f | flowers | Dianthus_superbus | Caryophyllaceae | Jamnigalm | 250 | 47.004; 13.112 | 30.07.2021; 10:03:34 | 1770 | 1730 | carbonate |
| 14l | leaves | Dianthus_superbus | Caryophyllaceae | Jamnigalm | 250 | 47.004; 13.112 | 30.07.2021; 10:03:34 | 1770 | 1730 | carbonate |
| 15f | flowers | Digitalis_grandiflora | Plantaginaceae | Jamnigalm | 254 | 47.002; 13.119 | 30.07.2021; - | 1770 | 1486 | carbonate |
| 15l | leaves | Digitalis_grandiflora | Plantaginaceae | Jamnigalm | 254 | 47.002; 13.119 | 30.07.2021; - | 1770 | 1486 | carbonate |
| 16f | flowers | Epilobium_angustifolium | Onagraceae | Jamnigalm | 254 | 47.002; 13.119 | 30.07.2021; - | 1770 | 1486 | carbonate |
| 16l | leaves | Epilobium_angustifolium | Onagraceae | Jamnigalm | 254 | 47.002; 13.119 | 30.07.2021; - | 1770 | 1486 | carbonate |
| 17f | flowers | Erigeron_annuus | Asteraceae | Turnersee | 235 | 46.579; 14.581 | 26.07.2021; 12:33:30 | 490 | 512 | carbonate |
| 17l | leaves | Erigeron_annuus | Asteraceae | Turnersee | 235 | 46.579; 14.581 | 26.07.2021; 12:33:30 | 490 | 512 | carbonate |
| 18f | flowers | Galium_mollugo | Rubiaceae | Sablatnighof | 242 | 46.571; 14.602 | 28.07.2021; 08:59:41 | 490 | 490 | carbonate |
| 18l | leaves | Galium_mollugo | Rubiaceae | Sablatnighof | 242 | 46.571; 14.602 | 28.07.2021; 08:59:41 | 490 | 490 | carbonate |
| 19f | flowers | Galium_verum | Rubiaceae | Sablatnighof | 242 | 46.571; 14.602 | 28.07.2021; 08:59:41 | 490 | 490 | carbonate |
| 19l | leaves | Galium_verum | Rubiaceae | Sablatnighof | 242 | 46.571; 14.602 | 28.07.2021; 08:59:41 | 490 | 490 | carbonate |
| 20f | flowers | Gentiana_bavarica | Gentanaceae | Moelltaler_Glacier | 246 | 47.015; 13.015 | 29.07.2021; 11:19:29 | 2225 | 2220 | silicate |
| 20l | leaves | Gentiana_bavarica | Gentanaceae | Moelltaler_Glacier | 246 | 47.015; 13.015 | 29.07.2021; 11:19:29 | 2225 | 2220 | silicate |
| 21f | flowers | Geranium_pratense | Geraniaceae | Zedlitzberg | 243 | 46.775; 14.066 | 28.07.2021; 13:27:02 | 900 | 900 | silicate |
| 21l | leaves | Geranium_pratense | Geraniaceae | Zedlitzberg | 243 | 46.775; 14.066 | 28.07.2021; 13:27:02 | 900 | 900 | silicate |
| 22f | flowers | Geranium_robertianum | Geraniaceae | Turnersee | 234 | 46.579; 14.582 | 26.07.2021; 12:09:07 | 490 | 500 | carbonate |
| 22l | leaves | Geranium_robertianum | Geraniaceae | Turnersee | 234 | 46.579; 14.582 | 26.07.2021; 12:09:07 | 490 | 500 | carbonate |
| 23f | flowers | Geum_montanum | Rosaceae | Moelltaler_Glacier | 246 | 47.015; 13.015 | 29.07.2021; 11:19:29 | 2225 | 2220 | silicate |
| 23l | leaves | Geum_montanum | Rosaceae | Moelltaler_Glacier | 246 | 47.015; 13.015 | 29.07.2021; 11:19:29 | 2225 | 2220 | silicate |
| 24f | flowers | Helianthemum_grandiflorum | Cistaceae | Petzen | 239 | 46.511; 14.769 | 27.07.2021; 11:41:48 | 1770 | 1860 | carbonate |
| 25f | flowers | Heliosperma_quadrifida | Caryophyllaceae | Petzen | 237 | 46.515; 14.772 | 27.07.2021; 10:24:08 | 1770 | 1750 | carbonate |
| 25l | leaves | Heliosperma_quadrifida | Caryophyllaceae | Petzen | 237 | 46.515; 14.772 | 27.07.2021; 10:24:08 | 1770 | 1750 | carbonate |
| 26f | flowers | Heracleum_austriacum | Apiaceae | Petzen | 237 | 46.515; 14.772 | 27.07.2021; 10:24:08 | 1770 | 1750 | carbonate |
| 26l | leaves | Heracleum_austriacum | Apiaceae | Petzen | 237 | 46.515; 14.772 | 27.07.2021; 10:24:08 | 1770 | 1750 | carbonate |
| 27f | flowers | Hypericum_maculatum | Hypericaceae | Jamnigalm | 250 | 47.004; 13.112 | 30.07.2021; 10:03:34 | 1770 | 1730 | carbonate |
| 27l | leaves | Hypericum_maculatum | Hypericaceae | Jamnigalm | 250 | 47.004; 13.112 | 30.07.2021; 10:03:34 | 1770 | 1730 | carbonate |
| 28f | flowers | Imaptiens_glandulifera | Balsaminaceae | Ebenthalerschlucht | 233 | 46.598; 14.355 | 25.07.2021; - | 490 | 450 | carbonate |
| 28l | leaves | Imaptiens_glandulifera | Balsaminaceae | Ebenthalerschlucht | 233 | 46.598; 14.355 | 25.07.2021; - | 490 | 450 | carbonate |
| 29f | flowers | Impatiens_parviflora | Balsaminaceae | Turnersee | 235 | 46.579; 14.581 | 26.07.2021; 12:33:30 | 490 | 512 | carbonate |
| 29l | leaves | Impatiens_parviflora | Balsaminaceae | Turnersee | 235 | 46.579; 14.581 | 26.07.2021; 12:33:30 | 490 | 512 | carbonate |
| 30f | flowers | Knautia_arvensis | Caprifoliaceae | Turnersee | 236 | 46.586; 14.582 | 26.07.2021; 15:22:35 | 490 | 512 | carbonate |
| 30l | leaves | Knautia_arvensis | Caprifoliaceae | Turnersee | 236 | 46.586; 14.582 | 26.07.2021; 15:22:35 | 490 | 512 | carbonate |
| 31f | flowers | Knautia_drymeia | Caprifoliaceae | Petzen | 238 | 46.513; 14.771 | 27.07.2021; 11:03:41 | 1770 | 1795 | carbonate |
| 31l | leaves | Knautia_drymeia | Caprifoliaceae | Petzen | 238 | 46.513; 14.771 | 27.07.2021; 11:03:41 | 1770 | 1795 | carbonate |
| 32f | flowers | Lotus_corniculatus | Fabaceae | Jamnigalm | 253 | 47.009; 13.100 | 30.07.2021; 13:07:08 | 1770 | 1880 | carbonate |
| 32l | leaves | Lotus_corniculatus | Fabaceae | Jamnigalm | 253 | 47.009; 13.100 | 30.07.2021; 13:07:08 | 1770 | 1880 | carbonate |
| 33f | flowers | Minuartia_austriaca | Caryophyllaceae | Petzen | 238 | 46.513; 14.771 | 27.07.2021; 11:03:41 | 1770 | 1795 | carbonate |
| 33l | leaves | Minuartia_austriaca | Caryophyllaceae | Petzen | 238 | 46.513; 14.771 | 27.07.2021; 11:03:41 | 1770 | 1795 | carbonate |
| 34f | flowers | Mycelis_muralis | Asteraceae | Turnersee | 234 | 46.579; 14.582 | 26.07.2021; 12:09:07 | 490 | 500 | carbonate |
| 34l | leaves | Mycelis_muralis | Asteraceae | Turnersee | 234 | 46.579; 14.582 | 26.07.2021; 12:09:07 | 490 | 500 | carbonate |
| 35f | flowers | Origanum_vulgare | Lamiaceae | Turnersee | 236 | 46.586; 14.582 | 26.07.2021; 15:22:35 | 490 | 512 | carbonate |
| 35l | leaves | Origanum_vulgare | Lamiaceae | Turnersee | 236 | 46.586; 14.582 | 26.07.2021; 15:22:35 | 490 | 512 | carbonate |
| 36f | flowers | Peucedanum_ostruthium | Apiaceae | Petzen | 238 | 46.513; 14.771 | 27.07.2021; 11:03:41 | 1770 | 1795 | carbonate |
| 36l | leaves | Peucedanum_ostruthium | Apiaceae | Petzen | 238 | 46.513; 14.771 | 27.07.2021; 11:03:41 | 1770 | 1795 | carbonate |
| 37f | flowers | Pimpinella_saxifraga | Apiaceae | Sablatnighof | 242 | 46.571; 14.602 | 28.07.2021; 08:59:41 | 490 | 490 | carbonate |
| 37l | leaves | Pimpinella_saxifraga | Apiaceae | Sablatnighof | 242 | 46.571; 14.602 | 28.07.2021; 08:59:41 | 490 | 490 | carbonate |
| 38f | flowers | Potentilla_aurea | Rosaceae | Moelltaler_Glacier | 246 | 47.015; 13.015 | 29.07.2021; 11:19:29 | 2225 | 2220 | silicate |
| 38l | leaves | Potentilla_aurea | Rosaceae | Moelltaler_Glacier | 246 | 47.015; 13.015 | 29.07.2021; 11:19:29 | 2225 | 2220 | silicate |
| 39f | flowers | Ranunculus_acris | Ranunculaceae | Zedlitzberg | 243 | 46.775; 14.066 | 28.07.2021; 13:27:02 | 900 | 900 | silicate |
| 39l | leaves | Ranunculus_acris | Ranunculaceae | Zedlitzberg | 243 | 46.775; 14.066 | 28.07.2021; 13:27:02 | 900 | 900 | silicate |
| 40f | flowers | Ranunculus_glacialis | Ranunculaceae | Moelltaler_Glacier | 249 | 47.031; 13.009 | 29.07.2021; 15:16:01 | 2225 | 2760 | silicate |
| 40l | leaves | Ranunculus_glacialis | Ranunculaceae | Moelltaler_Glacier | 249 | 47.031; 13.009 | 29.07.2021; 15:16:01 | 2225 | 2760 | silicate |
| 41f | flowers | Rhododendron_ferrugineum | Ericaceae | Moelltaler_Glacier | 247 | 47.015; 13.014 | 29.07.2021; 11:54:32 | 2225 | 2250 | silicate |
| 41l | leaves | Rhododendron_ferrugineum | Ericaceae | Moelltaler_Glacier | 247 | 47.015; 13.014 | 29.07.2021; 11:54:32 | 2225 | 2250 | silicate |
| 42f | flowers | Rhododendron_hirsutum | Ericaceae | Petzen | 237 | 46.515; 14.772 | 27.07.2021; 10:24:08 | 1770 | 1750 | carbonate |
| 42l | leaves | Rhododendron_hirsutum | Ericaceae | Petzen | 237 | 46.515; 14.772 | 27.07.2021; 10:24:08 | 1770 | 1750 | carbonate |
| 43f | flowers | Salvia_pratensis | Lamiaceae | Turnersee | 236 | 46.586; 14.582 | 26.07.2021; 15:22:35 | 490 | 512 | carbonate |
| 43l | leaves | Salvia_pratensis | Lamiaceae | Turnersee | 236 | 46.586; 14.582 | 26.07.2021; 15:22:35 | 490 | 512 | carbonate |
| 44f | flowers | Saxifraga_moschata | Saxifragaceae | Moelltaler_Glacier | 245 | 47.015; 13.017 | 29.07.2021; 10:50:50 | 2225 | 2175 | silicate |
| 44l | leaves | Saxifraga_moschata | Saxifragaceae | Moelltaler_Glacier | 245 | 47.015; 13.017 | 29.07.2021; 10:50:50 | 2225 | 2175 | silicate |
| 45f | flowers | Silene_acaulis | Caryophyllaceae | Petzen | 240 | 46.505; 14.766 | 27.07.2021; 14:34:12 | 2225 | 2000 | carbonate |
| 45l | leaves | Silene_acaulis | Caryophyllaceae | Petzen | 240 | 46.505; 14.766 | 27.07.2021; 14:34:12 | 2225 | 2000 | carbonate |
| 46f | flowers | Trifolium_badium | Fabaceae | Moelltaler_Glacier | 248 | 47.016; 13.013 | 29.07.2021; 12:23:57 | 2225 | 2270 | silicate |
| 46l | leaves | Trifolium_badium | Fabaceae | Moelltaler_Glacier | 248 | 47.016; 13.013 | 29.07.2021; 12:23:57 | 2225 | 2270 | silicate |
| 47f | flowers | Trifolium_pratense | Fabaceae | Zedlitzberg | 243 | 46.775; 14.066 | 28.07.2021; 13:27:02 | 900 | 900 | silicate |
| 47l | leaves | Trifolium_pratense | Fabaceae | Zedlitzberg | 243 | 46.775; 14.066 | 28.07.2021; 13:27:02 | 900 | 900 | silicate |
| 48f | flowers | Trifolium_repens | Fabaceae | Zedlitzberg | 243 | 46.775; 14.066 | 28.07.2021; 13:27:02 | 900 | 900 | silicate |
| 48l | leaves | Trifolium_repens | Fabaceae | Zedlitzberg | 243 | 46.775; 14.066 | 28.07.2021; 13:27:02 | 900 | 900 | silicate |
| 49f | flowers | Verbascum_austriacum | Scrophulariaceae | Turnersee | 236 | 46.586; 14.582 | 26.07.2021; 15:22:35 | 490 | 512 | carbonate |
| 49l | leaves | Verbascum_austriacum | Scrophulariaceae | Turnersee | 236 | 46.586; 14.582 | 26.07.2021; 15:22:35 | 490 | 512 | carbonate |
| 50f | flowers | Viola_bilflora | Violaceae | Moelltaler_Glacier | 245 | 47.015; 13.017 | 29.07.2021; 10:50:50 | 2225 | 2175 | silicate |
| 50l | leaves | Viola_bilflora | Violaceae | Moelltaler_Glacier | 245 | 47.015; 13.017 | 29.07.2021; 10:50:50 | 2225 | 2175 | silicate |

| Flowers - Leaves | Plant compartment | | | |
| --- | --- | --- | --- | --- |
|  | Bacteria | | Fungi | |
|  | P | R² | P | R² |
| Species richness | <0.001 | 0.234 | <0.001 | 0.286 |
| Shannon diversity | <0.001 | 0.412 | <0.001 | 0.115 |
| Abundance | 0.950 | <0.001 | 0.874 | <0.001 |
| Community Composition | 0.001 | 0.069 | 0.001 | 0.037 |

**Supplementary Table 2a: Comparison of microbial diversity, abundance, and community composition between flowers and leaves (p-values and R-squared values)**

*Statistically significant p-values (p < 0.05) are highlighted in red.*

**Supplementary Table 2b: Comparison of microbial diversity, abundance, and community composition across geographical locations, soil types, altitudes, and plant families (p-values and R-squared values)**

| Flowers | Locations | | | | Soil Types | | | | Altitude | | | | Family Identity | | | |
| --- | --- | --- | --- | --- | --- | --- | --- | --- | --- | --- | --- | --- | --- | --- | --- | --- |
|  | Bacteria | | Fungi | | Bacteria | | Fungi | | Bacteria | | Fungi | | Bacteria | | Fungi | |
|  | P | R² | P | R² | P | R² | P | R² | P | R² | P | R² | P | R² | P | R² |
| Species richness | 0.025 | 0.206 | 0.003 | 0.338 | 0.625 | <0.001 | 0.420 | 0.086 | 0.381 | <0.001 | 0.004 | 0.251 | 0.038 | 0.285 | 0.091 | 0.280 |
| Shannon diversity | 0.166 | 0.151 | 0.001 | 0.306 | 0.270 | 0.011 | 0.724 | 0.008 | 0.040 | 0.065 | <0.004 | 0.192 | 0.462 | 0.151 | 0.003 | 0.408 |
| Abundance | 0.223 | 0.104 | 0.412 | 0.099 | 0.435 | 0.005 | 0.077 | 0.006 | 0.133 | 0.026 | 0.309 | 0.001 | < 0.001 | 0.693 | 0.005 | 0.396 |
| Community Composition | 0.001 | 0.150 | 0.001 | 0.193 | 0.001 | 0.052 | 0.001 | 0.075 | 0.001 | 0.058 | 0.001 | 0.085 | 0.091 | 0.245 | 0.075 | 0.252 |
| Leaves | Bacteria | | Fungi | | Bacteria | | Fungi | | Bacteria | | Fungi | | Bacteria | | Fungi | |
|  | P | R² | P | R² | P | R² | P | R² | P | R² | P | R² | P | R² | P | R² |
| Species richness | 0.138 | 0.151 | 0.041 | 0.236 | 0.650 | <0.001 | 0.855 | 0.099 | 0.719 | <0.001 | 0.165 | 0.021 | 0.740 | 0.112 | 0.687 | 0.123 |
| Shannon diversity | 0.051 | 0.223 | <0.001 | 0.470 | 0.460 | 0.037 | 0.707 | 0.034 | 0.998 | <0.001 | 0.002 | 0.174 | 0.291 | 0.207 | 0.481 | 0.139 |
| Abundance | 0.333 | 0.111 | 0.221 | 0.141 | 0.308 | <0.001 | 0.259 | 0.011 | 0.204 | 0.014 | 0.247 | 0.008 | < 0.001 | 0.651 | <0.001 | 0.656 |
| Community Composition | 0.001 | 0.184 | 0.001 | 0.250 | 0.002 | 0.042 | 0.001 | 0.064 | 0.001 | 0.09 | 0.001 | 0.115 | 0.091 | 0.239 | 0.178 | 0.236 |

*Statistically significant p-values (p < 0.05) are highlighted in red*


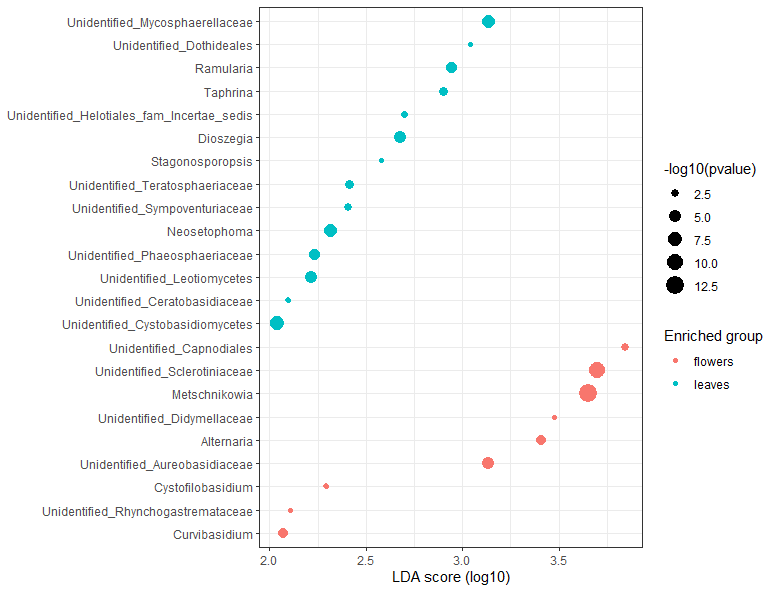


1. **Fungi**


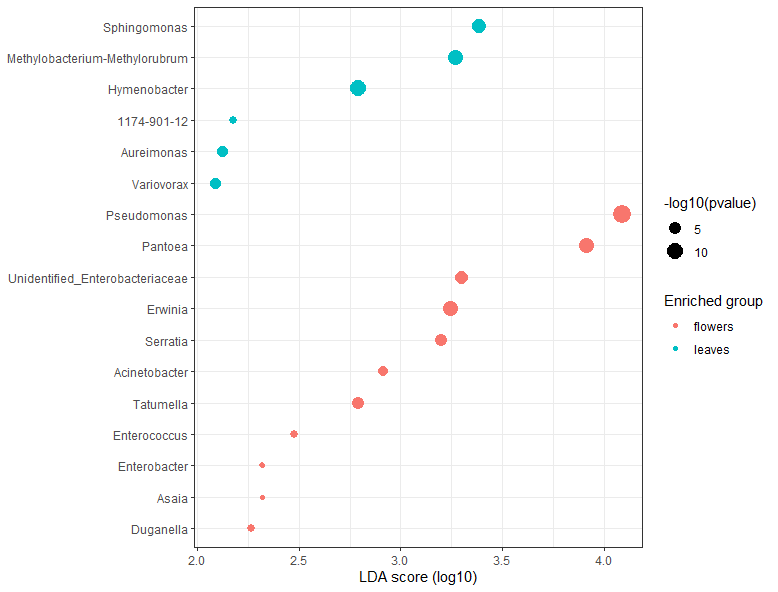


1. **Bacteria**


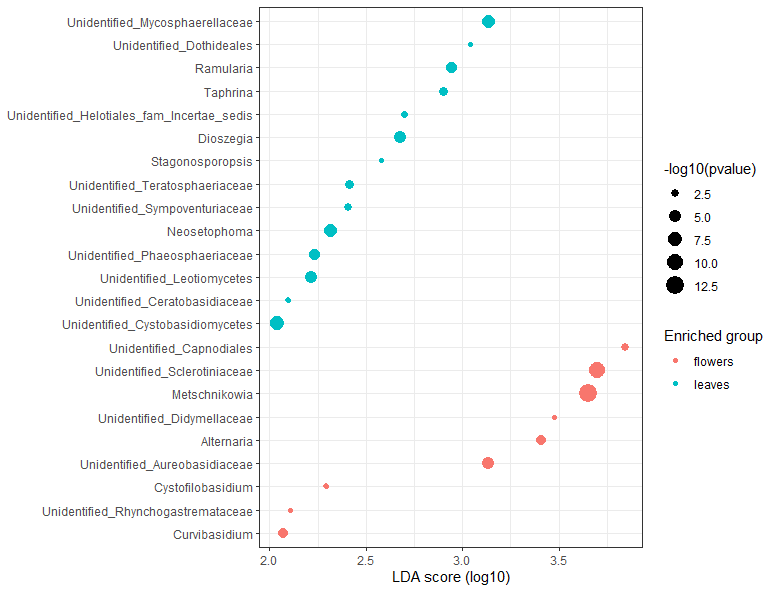


***Fungi***

**Supplementary Figure 1: Linear discriminant analysis Effect Size (LefSe) plots was used to identify significantly different genera between leaves and flowers (P-value cutoff = 0.05; Log LDA Score = 2).**


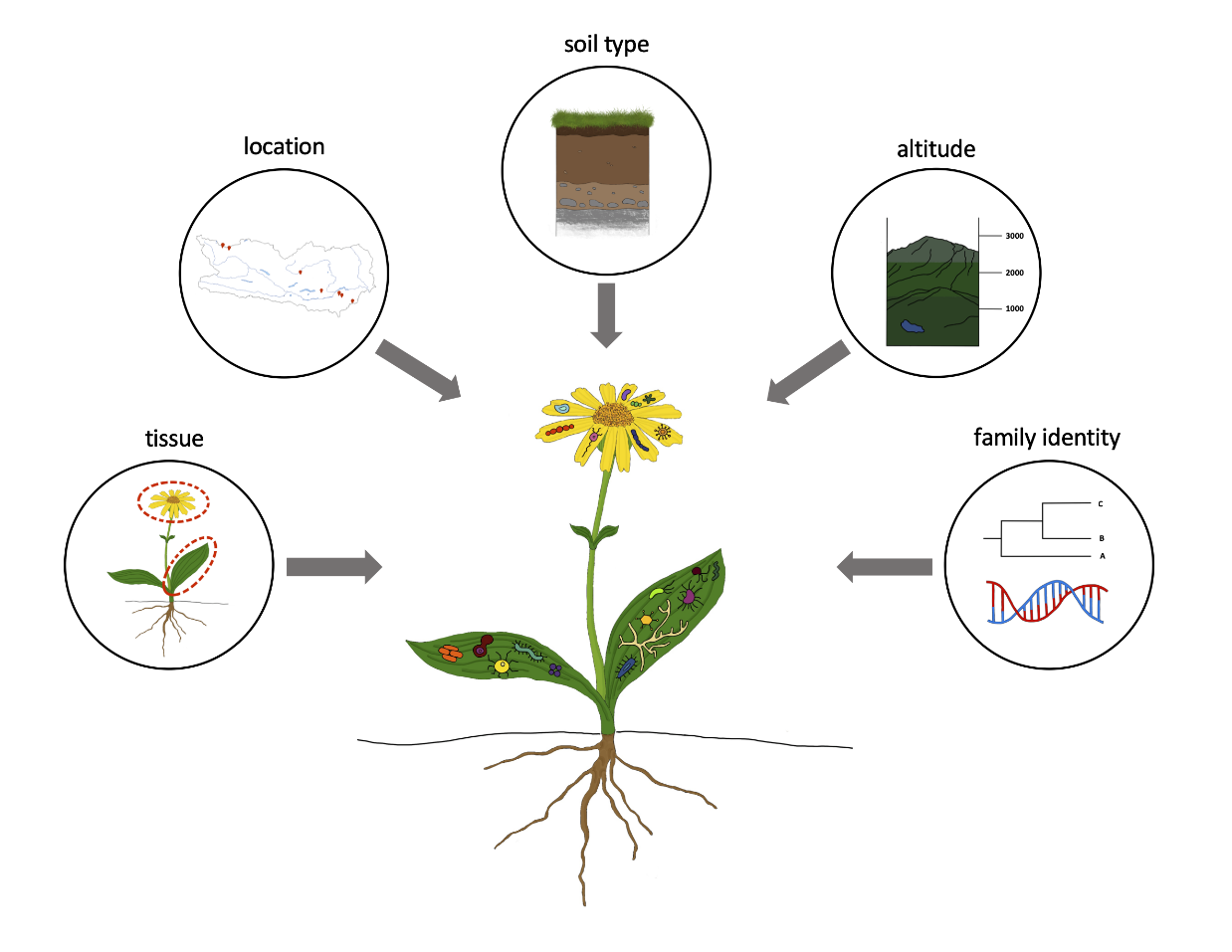


***Supplementary Figure 2: Conceptual framework illustrating the factors investigated in this study. The figure depicts the potential influence of various parameters (plant compartment, location, soil type, altitude, and family identity) on the plant microbiome.***
